# Risk Factors for Human Infection with Avian Influenza A H5N1, Vietnam, 2004

**DOI:** 10.3201/eid1212.060829

**Published:** 2006-12

**Authors:** Pham Ngoc Dinh, Hoang Thuy Long, Nguyen Thi Kim Tien, Nguyen Tran Hien, Le Thi Quynh Mai, Le Hong Phong, Le Van Tuan, Hoang Van Tan, Nguyen Binh Nguyen, Phan Van Tu, Nguyen Thi Minh Phuong, the World Health Organization/Global Outbreak Alert and Response Network Avian Influenza Investigation Team in Vietnam

**Affiliations:** * National Institute of Hygiene and Epidemiology, Hanoi, Vietnam;; † Pasteur Institute, Ho Chi Minh City, Vietnam

**Keywords:** Influenza A virus, H5N1 subtype, case-control studies, risk factor, human, Viet Nam, research

## Abstract

To evaluate risk factors for human infection with influenza A subtype H5N1, we performed a matched case-control study in Vietnam. We enrolled 28 case-patients who had laboratory-confirmed H5N1 infection during 2004 and 106 age-, sex-, and location-matched control-respondents. Data were analyzed by matched-pair analysis and multivariate conditional logistic regression. Factors that were independently associated with H5N1 infection were preparing sick or dead poultry for consumption <7 days before illness onset (matched odds ratio [OR] 8.99, 95% confidence interval [CI] 0.98–81.99, p = 0.05), having sick or dead poultry in the household <7 days before illness onset (matched OR 4.94, 95% CI 1.21–20.20, p = 0.03), and lack of an indoor water source (matched OR 6.46, 95% CI 1.20–34.81, p = 0.03). Factors not significantly associated with infection were raising healthy poultry, preparing healthy poultry for consumption, and exposure to persons with an acute respiratory illness.

## Abstract 2

TOC Summary: Risk factors include direct or household contact with sick or dead poultry and absence of an indoor water source.

The first indication that the current epizootic of highly pathogenic avian influenza subtype H5N1 (influenza A H5N1) would have a serious effect on human heath occurred in early 2004, when influenza H5N1 was identified in a series of patients admitted to the National Pediatric Hospital in Hanoi with severe viral pneumonia (*1*). Since then, large-scale and global spread of the disease in poultry has been accompanied by sporadic cases in humans. Despite many millions of avian infections and >200 human cases, knowledge of influenza H5N1 remains inadequate. Neither how these viruses are transmitted to humans nor, consequently, the most effective way to reduce the risk for infection is fully understood. Descriptive and analytic epidemiologic studies conducted in Hong Kong Special Administrative Region, People's Republic of China, during the 1997 outbreak of influenza H5N1 (*2**–**5*) identified visiting a live bird market as a risk factor. However, the current outbreak encompasses different viruses and different sociodemographic, farming, and behavioral contexts. Several seroprevalence studies of healthcare workers and a case-control study from Thailand have been published from the current outbreak (*6**–**9*), but further work is needed to develop and test hypotheses on the mechanism of transmission of influenza H5N1 to humans. To clarify the source and mode(s) of transmission of influenza H5N1 to humans and to guide the control and prevention of influenza, we conducted a case-control study of all cases of avian influenza H5N1 identified in humans in Vietnam in 2004.

## Materials and Methods

All persons with laboratory-confirmed influenza A H5 cases detected in Vietnam from January 1 through December 31, 2004, were eligible for enrollment as case-patients. Case-patients were identified from persons hospitalized with an acute respiratory infection considered by clinicians, on the basis of clinical and epidemiologic findings, to have a suspected case of H5N1 infection. Clinicians did not use a systematic case definition or screening protocol to identify patients eligible for testing for H5N1 infection. Throat swabs or tracheal aspirate samples were sent to the National Institute of Hygiene and Epidemiology in Hanoi or to the Pasteur Institute in Ho Chi Minh City for reverse transcription (RT) PCR and viral isolation. When possible, samples with positive results for influenza A H5 were sent to a World Health Organization (WHO) reference laboratory for confirmatory diagnosis.

For each case-patient, 4 control-respondents, individually matched by gender, age (age difference <24 months), and place of residence (same ward or village), were selected by use of a random number table from a list of persons fitting the selection criteria provided by the community health station near each case-patient's place of residence. Potential control-respondents were excluded if they reported having suffered an illness with respiratory symptoms and fever (temperature >38°C) during their matched case-patient's period of illness (onset to recovery or death). If the selected control-respondent refused to participate or did not meet the inclusion criteria, the geographically closest eligible person was then selected from the list. All eligible control-respondents were asked to provide a throat swab and venous blood (5 mL) to confirm that they were not currently or had not previously been infected with influenza A H5. Participation of case-patients and control-respondents was voluntary and required written consent and, for those aged <18 years, signature of a parent or guardian.

Trained interviewers administered a structured questionnaire to case-patients and control-respondents. If the case-patient or control-respondent was a child or if the case-patient had died, the questionnaire was administered to a proxy, usually the child's parent or a close family member living in the same household. The questionnaire collected information about demographic characteristics; preexisting health status; smoking behavior; potential animal, human, and environmental exposures to influenza A H5; and personal and household hygienic practices. Case-patients or their proxies were asked about exposures in the 7 days before illness onset, and control-respondents were asked about exposures during the same 7-day period as their matched case-patient.

### Definitions

Persons who met any of the following criteria were considered to be laboratory-confirmed influenza A H5 case-patients: 1) influenza A H5–specific RNA detected in a single specimen by RT-PCR by using 2 different primer pairs; 2) influenza A H5 detected in a single specimen by RT-PCR identification and by sequencing or virus isolation; 3) influenza A H5–specific RNA detected by RT-PCR in 2 different specimen types (e.g., throat swab and tracheal aspirate); and 4) influenza A H5–specific RNA detected by RT-PCR in 2 samples taken on different days. Control-respondents were considered to be true control-respondents if throat swab specimens were negative for influenza A H5–specific RNA by RT-PCR and anti-H5 antibodies could not be detected in serum samples by microneutralization assay (*10*).

### Laboratory Methods

Influenza A H5 subtype–specific RNA was detected in clinical samples by RT-PCR with primers that targeted regions of the hemagglutinin gene of the influenza H5N1 virus developed by WHO, the US Centers for Disease Control and Prevention (CDC), and the Government Virus Unit in Hong Kong. Clinical specimens were injected into Madin-Darby canine kidney cells for virus isolation, and RT-PCR was used to identify influenza A H5. Specimens and cell cultures suspected of containing influenza A H5 were handled according to recognized biosafety standards.

Serum samples were immediately processed, stored at –25°C, and shipped frozen on dry ice to CDC. To measure influenza A H5–specific antibody, microneutralization assay was conducted as previously described (*10*) by using H5N1 viruses A/Vietnam/1194/2004 and A/Vietnam/3212/2004. Microneutralization test results were considered to be positive if an anti-H5 titer of >40 was obtained by 2 independent assays.

### Statistical Analysis

Data entry and analysis of individual explanatory variables was performed by using Epi-Info 6 (CDC, Atlanta, GA, USA). Mantel-Haenszel matched-pair analysis (McNemar test) was used to estimate the strength and statistical significance of associations between exposures and influenza A H5 infection. An association was considered statistically significant if 2-sided tests of significance had a p-value <0.05. To examine independence of effects, multivariate conditional logistic regression was performed by using the conditional logistical regression (CLogit) function in Stata/SE 8.0 for Windows (Stata Corp LP, College Station, TX, USA). Any variables with p<0.2 after matched analysis were included in the initial model. A backward stepwise variable- selection strategy was used to construct a final model with a significance level of >0.1 for removal and a significance level of <0.05 for re-entry into the model. Persons missing data for variables under study were excluded from any analysis involving the missing variable. Collinearity was assessed by generating a correlation coefficient matrix for all variables to be considered for inclusion in the regression model. The presence of effect-measure modification by age and sex was assessed for all variables in the final model by entering product terms. A final model was achieved by entering the variables retained in the backward selection model.

The attributable risk percent (AR%) was estimated as follows: ([odds ratio (OR) – 1]/OR) ×100. The population attributable risk percent (PAR%) was estimated as follows: PAR% = AR% × proportion of case-patients exposed.

## Results

A total of 28 laboratory-confirmed influenza A H5 cases were detected in 2004 from 15 provinces of Vietnam; 21 (75%) were fatal. All 28 cases were RT-PCR positive for influenza A subtype H5 at either the National Institute of Hygiene and Epidemiology, Hanoi, or the Pasteur Institute, Ho Chi Minh City; H5N1 virus was isolated in 12. The diagnosis of influenza A H5 infection was independently confirmed for 25: 20 at a WHO reference laboratory and 5 at the Oxford University Clinical Research Unit in Vietnam.

The interviews began on February 7, 2004, at which time 20 of the 28 case-patients had already been identified. The interval between onset of illness and interview was a mean of 35.7 days; the maximum interval was 63 days. The mean age of case-patients was 14 years (range 1–31 years, median 15 years), and 9 (32%) patients were children <10 years of age. The numbers of male and female case-patients were equal (14 each). Among confirmed case-patients were 2 family clusters (mother and daughter and 2 sisters). A total of 106 control-respondents were enrolled, 4 per case-patient, except for 3 case-patients aged <5 years for whom only 1, 2, and 3 control-respondents could be recruited per case, respectively. All control-respondents were negative for avian influenza A H5–specific RNA by RT-PCR and for anti-H5 antibodies by microneutralization assay. None of the case-patients or control-respondents worked in the commercial (industrial) poultry-raising sector.

The results of matched-pair analysis are shown in Table 1. Direct handling of sick or dead poultry in the 7 days before onset of illness had the strongest point estimate of effect (matched OR 31) and high statistical significance (p<0.001) despite wide confidence limits (95% confidence interval [CI] 3.4–1150). The presence of sick or dying poultry in the household (matched OR 7.4, 95% CI 2.7–59) or neighborhood (matched OR 3.9, 95% CI 1.0–55.7) was also statistically associated with infection as was the absence of an indoor water source in the household (matched OR 5.0, 95% CI 1.3–77.0) and education to high school level or higher (matched OR 16.0, 95% CI 1.2–594.1).

**Table 1:** Matched-pair analysis of potential risk factors for human infection with avian influenza A H5N1, Vietnam, 2004

Exposure and characteristics	Case-patients (n = 28), n (%)	Control-respondents (n = 106), n (%)	Matched OR* (95% CI)	p value
High school, college, or university education (persons >14 y of age)	8 (53)†	17 (29)‡	16.0 (1.2–594.1)	0.03
Family size >5 persons	8 (29)	32 (30)	1.2 (0.4–4.0)	0.88
Ever smoked	3 (11)	10 (9)	2.0 (0.1–30.5)	0.91
Chronic medical conditions	3 (11)	9 (8)	1.3 (0.2–7.7)	0.93
Poultry-related exposures§				
Prepared and cooked healthy poultry	9 (32)	24 (23)	2.2 (0.6–10.4)	0.249
Prepared and cooked sick or dead poultry	9 (32)	6 (6)	31.0 (3.4–1150)	<0.001
Helped prepare or cook sick or dead poultry	7 (25)	12 (11)	2.6 (0.8–8.7)	0.102
Bought live poultry for household consumption	3 (11)	9 (8)	1.2 (0.2–7.0)	0.895
Bought freshly killed poultry for household consumption	0 (0)	11 (10)	Incalculable	-
Live poultry in household	18 (64)	52 (49)	3.0 (0.9–10.0)	0.103
Sick or dead poultry in household	15 (54)	20 (19)	7.4 (2.7–59.0)	<0.001
Live poultry in neighborhood	19 (79)¶	75 (74)#	1.07 (0.2–6.6)	0.810
Sick or dead poultry in neighborhood	12 (43)	29 (27)	3.9 (1.0–55.7)	0.05
Farm or family with >150 poultry within 100 m	4 (14)	16 (15)	1.0 (0.2–4.2)	0.742
Household members work with commercial poultry	1 (4)	2 (2)	2.0 (0.0–38.4)	0.88
Other animal-related exposures				
Pigs in household	9 (32)	28 (26)	1.4 (0.3–6.4)	0.838
Pig in neighborhood	15 (54)	48 (45)	2.0 (0.5–7.2)	0.505
Dogs in household	18 (64)	58 (55)	1.7 (0.6–4.7)	0.430
Cats in household	9 (32)	23 (22)	2.0 (0.6–5.9)	0.374
Buffalo in household	1 (4)	1 (1)	4.0 (0.1–314)	0.86
Cows in household	5 (18)	14 (13)	2.4 (0.3–17.4)	0.581
Human-related exposures§				
Exposed to patients with acute respiratory infection (temperature ≥38°C)	6 (21)	11 (10)	2.4 (0.7–13.5)	0.145
Exposed to hospitalized patients with acute respiratory infection	5 (18)	9 (8)	2.4 (0.6–12.9)	0.210
Hygiene- and environment-related exposures				
Handwashing before eating (usually or sometimes)	23 (82)	90 (85)	1.3 (0.3–5.6)	0.911
Handwashing >3 times/d	25 (89)	87 (82)	0.53 (0.1–2.4)	0.568
Wading in ponds, rice fields, ditches	4 (14)	7 (7)	2.4 (0.4–19.2)	0.469
No indoor water source in household	22 (79)	64 (60)	5.0 (1.3–77.0)	0.024
Poor hygiene conditions**	12 (43)	47 (44)	1.0 (0.3–3.4)	0.829

*Matched analysis using McNemar (Mantel-Haenszel) test statistics; 95% confidence limits are exact intervals for maximum likelihood estimate; OR, odds ratio; CI, confidence interval. †n = 15. ‡n = 58. §7 d before illness onset in case-patient. ¶n = 24. #n = 102. **A composite measure of 7 indicators: dust level in person’s home, type of flooring, frequency of house cleaning, habit of washing hands before eating, habit of washing fruit before eating, estimated frequency of handwashing/d, and interviewer’s assessment of household cleanliness.

Eight variables with p<0.2 were considered for inclusion in the conditional logistic regression model to estimate independence of effects. Although significantly associated with infection in the single-variable analysis, the presence of sick or dead poultry in the neighborhood was excluded from the final regression model because missing data for this variable led to the exclusion of 36 participants (6 case-patients and 30 control-respondents). Educational level was excluded because it was not a relevant variable for the 13 case-patients <15 years of age. Because of the 2 family clusters, each comprising 2 case-patients, the influence of clustering of household-level factors on the regression model was investigated by running the regression model first with all cases and then again including only 1 case from each of these 2 households. All 4 variations of 1 case from each household were run. Because the outcomes of these different approaches did not differ, all cases were included in the final model.

The final conditional logistic regression model included 3 variables as independent risk factors for H5N1 infection (Table 2). Of the 28 case-patients, 16 (57%) had either sick or dead poultry in their household or had directly prepared sick or dead poultry for consumption; another 6 reported sick or dead poultry in the neighborhood. Of the 28 case-patients, 22 (79%) did not have an indoor water source. No statistically significant effect-measure modification was detected.

**Table 2:** Results of multivariate analysis of potential risk factors for human infection with avian influenza A H5N1, Vietnam, 2004*

Exposure and characteristics	Odds ratio	95% CI	p value
Prepare and cook sick or dead poultry	8.99	0.98–81.99	0.052
Sick or dead poultry in household	4.94	1.21–20.20	0.026
No indoor water source in household	6.46	1.20–34.81	0.03

*Conditional logistic regression; final model with 3 variables entered; χ^2^ for likelihood ratio test = 28.35; p<0.001; no. observations = 134; CI, confidence interval.

Among persons who prepared sick or dead poultry for consumption, the proportion of H5N1 cases attributable to this practice (AR%) is estimated in this study to be 89% ([(8.99 – 1)/8.99] × 100). However, because only 32% of all case-patients reported this practice, stopping this practice would prevent only an estimated 28% of H5N1 cases (PAR% = 0.89 × 0.32).

## Discussion

### Source of Infection

This study identified the presence in the household and the handling of dead or sick poultry in an H5N1-affected area as risk factors for human H5N1 infection. Although not surprising, these findings reinforce the hypothesis that close contact with infected domestic poultry is the primary source of transmission of influenza H5N1 to humans. The absence of a statistical association between infection and contact with other animals such as pigs, cats, or dogs is reassuring. Replication and excretion of H5N1 by asymptomatic domestic waterfowl has been demonstrated and is a plausible source of infection for humans (*11*); however, although a 1997 case-control study found that visiting live poultry markets was a risk factor for human influenza A H5N1 infection (*2*), our study and the study from Thailand (*9*) did not identify contact with healthy poultry as a risk factor. However, viral titers in asymptomatic waterfowl may be much lower than in diseased poultry (*11*) and may therefore pose a low risk to humans, which our study was underpowered to identify.

Despite evidence that limited human-to-human transmission of H5N1 has occurred (*3**,**5**,**12*), we observed no significant differences between case-patients and control-respondents in terms of exposure to persons who might be a source of H5N1 infection, e.g., patients with an acute respiratory infection. This finding is consistent with that of the case-control study in Thailand (*9*).

## Route of Transmission

Our study found an association between infection and direct and household contact with diseased poultry. This association, if true, could operate by 2 mechanisms. First, transmission may be by inhalation or conjunctival deposition of large infectious droplets, which may travel only short distances (*13*); second, the presence of infected poultry in the home and preparation of infected poultry for consumption may result in exposure to higher virus concentrations than other types of exposure. An alternative hypothesis is that the consumption, rather than the preparation, of infected poultry is the route of infection. Domestic cats and possibly tigers have been infected by the oral route (*14**,**15*), and the observed association between preparation and infection could be the result of a confounding association between preparing an infected bird (apparent risk factor) and consuming an infected bird (true risk factor). Unfortunately, the study did not ask about consumption of sick or dead poultry because the gastrointestinal route of transmission was not considered plausible at the time the study was designed.

The unexpected finding that the absence of an indoor water source is associated with infection may point toward a role for self-inoculation into conjunctival, nasal, or oral mucosa by contaminated hands or possibly foodstuffs. Certainly, the environmental stability of avian influenza viruses is sufficient for this to occur (*16*), and handwashing has been shown to decrease risk for respiratory infections (*17*). However, we asked 2 questions specifically about handwashing behavior, and neither was significantly associated with infection (Table 1). An alternative, and more controversial, explanation is that people without access to an indoor water source may acquire infection by drinking or washing in outdoor water sources contaminated with feces from infected poultry. This hypothesis is plausible, given the extended survival of avian influenza viruses in water (*18**,**19*) and the demonstration of oral infectivity in cats (*14*). Although an indoor water source might simply be a proxy indicator of socioeconomic status or the priority the household gives to hygiene, all other hygiene factors and a composite hygiene index did not show a statistically significant association with infection.

That 5 case-patients did not report any exposure to sick poultry in the 7 days before illness onset has several possible explanations: recall bias by case-patients or their proxies, infection acquired from infected but asymptomatic animals such as ducks, an incubation period >7 days, or infection from a contaminated environment as discussed above.

### Study Limitations

A major source of potential bias in this and other studies of risk factors for human H5N1 infection is the use of self-reported prior exposure to sick poultry as a screening tool for identifying potential case-patients. Use of this tool introduces a selection bias that favors finding greater exposure to sick poultry among case-patients than other groups, regardless whether the relationship is causal. In our study, clinicians were not using a systematic screening tool to identify possible H5N1 case-patients, but knowledge of poultry as the source of human H5N1 infection was ubiquitous. Possibly, H5N1 case-patients who did not have exposure to sick poultry may have been less likely to be identified than case-patients who did report this exposure. However, 5 of the 28 case-patients (18%) did not report exposure to sick poultry, indicating that exposure to sick poultry was not a prerequisite for identification as a case-patient. In light of this conflict between clinical necessity and study purity, estimates of the size of the association between exposure to sick poultry and H5N1 infection could be interpreted as maximums that are likely to have been inflated by this selection bias.

The relatively small number of case-patients means that the study may be underpowered to detect factors posing only a moderate risk for infection and to detect effect modification. A standardized questionnaire and trained interviewing staff were used to try to minimize interviewer bias, but masking the interviewers as to the case or control status of the respondents was not possible. Recall bias was likely to have occurred, especially because the high case-fatality rate meant that a larger proportion of interviews in the case group (26/28) than the control group (35/106) were completed by proxies. The substantial delay between onset of illness and interviews (mean 35.7 days) is also a potential source of recall bias.

The finding of a significant positive association between level of education and risk for infection was unexpected and is difficult to explain. It may be the consequence of a bias introduced by proxy respondents for deceased case-patients reporting higher levels of education than case-patients had actually achieved.

Misclassification of case-patients and control-respondents was unlikely. All control-respondents were demonstrated to have no detectable antibodies to H5N1, and all case-patients had a clinically compatible illness with laboratory evidence of H5N1 infection, which was independently verified for 25 (89%) of the 28 cases.

### Public Health and Research Implications

Preparing sick or dead poultry for consumption in an H5N1-affected area is a risky practice. Although this study cannot estimate the absolute risk, among those who prepared sick or dead poultry for consumption, a high proportion of infections could be attributed to this practice. However, as the practice was not that widespread in our study participants, stopping it would prevent only an estimated 28% of H5N1 cases. Less risky but more widespread practices probably account for a greater proportion of H5N1 cases; these practices must also be identified and tackled. Regardless whether consumption of infected poultry is itself a risk factor, preparation and consumption of sick or dead poultry in infected areas must stop. That all 106 persons selected as control-respondents from communities with at least 1 confirmed human H5N1 case were negative for H5N1 antibodies adds further evidence to the belief that widespread subclinical H5N1 infection has not yet occurred in Southeast Asia (*20*).

The finding of an association between lack of access to an indoor water source and H5N1 infection provides an interesting basis for formulating new hypotheses, but it is not sufficiently strong evidence for concluding that H5N1 transmission is occurring by water or as a result of inadequate hygiene. Despite 2 reports of exposure to potentially contaminated water in Vietnamese H5N1 case-patients ([21]; pers. comm., Ministry of Health, Vietnam), no human cases of H5N1 infection have been directly attributed to exposure to contaminated water. Nevertheless, hygiene practices and access to safe water have collateral benefits regardless of H5N1 and should be encouraged and pursued. Environmental investigations are needed to sample water sources in and around the households of incident H5N1 case-patients and compare the findings to water sources sampled in and around unaffected households.

Familial clusters of cases have been a significant feature of the epidemiology of H5N1 infection since 2004 in that numerous clusters have occurred in Vietnam, Thailand, Cambodia, Indonesia, and Turkey (*22**,**23*). Although common exposures and behavior may be one explanation for the marked clustering, most clusters have involved blood relatives such as sibling pairs or parent-child groups rather than unrelated pairs such as husbands and wives. This finding suggests that inherited biologic factors, such as sialic acid receptor phenotype or immune response, may be determinants of infection and disease. Studying intrinsic determinants of susceptibility will require pooling of data and samples from affected families across affected countries. If intrinsic susceptibility were a risk determinant, it might dilute associations between certain behavior and infection unless the analyses were undertaken within subgroups that are homogeneous with respect to their intrinsic susceptibility. In this respect, intrafamilial studies that combine measures of biologic susceptibility with data about behavioral patterns, including food consumption and hygiene practices, may be particularly enlightening.
